# Herbal medicine (Suoquan) for treating nocturnal enuresis: A protocol for a systematic review of randomized controlled trials

**DOI:** 10.1097/MD.0000000000010391

**Published:** 2018-04-27

**Authors:** Yoo Been Lee, Ju Ah Lee, Soobin Jang, Hye Lim Lee

**Affiliations:** a Department of Pediatrics, College of Korean Medicine; b Department of Internal Medicine, College of Korean Medicine, Gachon University, Seongnam; c Clinical Medicine Division, Korea Institute or Oriental Medicine, Daejeon, South Korea.

**Keywords:** Chukchunwhan, herbal medicine, nocturnal enuresis, protocol, Suoquan, systematic review

## Abstract

**Background:**

Suoquan is widely used to treat frequent urination, enuresis, and other diseases caused by kidney qi deficiency. Many clinical trials assessing the efficacy and safety of Suoquan for the treatment of enuresis have been reported. This review will assess the clinical evidence for and against the use of Suoquan as a treatment for enuresis.

**Methods and analysis:**

Fourteen databases will be searched until 2018. We will include randomized controlled trials (RCTs) examining Suoquan decoctions for any type of enuresis. All RCTs of decoctions or modified decoctions will be included. The methodological qualities of the RCTs will be assessed using the Cochrane Collaboration tool for assessing risk of bias.

**Ethics and dissemination:**

This systematic review will be published in a peer-reviewed journal. The review will be disseminated both electronically and in print. It will be updated to inform and guide healthcare practices.

**Trial registration number:**

CRD42018087900

## Abstract 2

Supplemental Digital Content is available in the text

## 1 Introduction

Nocturnal enuresis (NE) refers to involuntary urination at night from 5 years of age or older.^[1]^ Enuresis can be classified as monosymptomatic enuresis nocturna (MEN) or nonmonosymptomatic enuresis nocturna (NMEN). MEN refers to urinating only at night without other bladder dysfunction, whereas NMEN includes dysfunction of the lower urinary tract with or without daytime incontinence.^[2]^

The prevalence of NE in different age groups is similar worldwide. Approximately 10% of 7-year-old children have NE, every year 15% is treated by itself, and it is known to affect only 1% to 2% of adults. The male-to-female ratio is 1.5:1.^[3]^ NE affects the health-related quality of life (HRQoL) of both child patients and their families.^[4]^ Research has shown that children with NE have a poor self-image and lower self-esteem than their peers.^[5]^ Furthermore, NE can cause psychosocial dysfunction; between 20% and 30% of children with NE fulfill the criteria for psychiatric disorders in the International Classification of Diseases 10th Revision or Diagnostic and Statistical Manual of Mental Disorders IV.^[6]^

The current understanding of the mechanism of NE is initial reduced secretion of antidiuretic hormones during sleep, followed by problems with bladder function and then malfunctions in the mechanism that causes the brain to wake up when the bladder is full.^[7]^ In traditional Korean medicine (TKM), NE is associated with the lung, kidney, spleen, and bladder. The failure of the bladder's retentive power is thought to be caused by kidney qi deficiency, vacuity cold of the lower origin, qi deficiency of the spleen and lung, or bladder damp-heat.^[8]^ Generally accepted treatments are oral pharmacological and behavioral therapies.^[9]^ The preferred behavioral treatment involves a bed alarm.^[10]^ If medication is stopped, the recurrence rate is high. Parents are also reluctant to take medication for a long time as a result of possible side effects.^[11]^ Therefore, a large number of patients with NE seek complementary and alternative approaches. Studies have recently been conducted on moxibustion,^[12]^ acupuncture,^[13]^ psychotherapy, and chiropractic^[14]^ and herbal medicine^[8,15]^ as a treatment for NE. However, no systematic reviews of the effects of Suoquan formulas on NE have been published.

In this review, we will investigate evidence related to the effectiveness of Suoquan formulas, which are widely used in TKM and traditional Chinese medicine (TCM) for treating the symptoms of NE.

## 2 Methods

### 2.1 Study registration

This study will follow the guidelines outlined in the Preferred Reporting Items for Systematic Reviews and Meta-Analyses (PRISMA) statement for meta-analyses of healthcare interventions;^[16]^ additionally, the current protocol report adheres to the PRISMA Protocols.^[17]^ The protocol for this systematic review has been registered on PROSPERO 2018 under the number CRD42018087900.

### 2.2 Ethic approval

This is not a clinical study. Therefore, ethic approval is not needed.

### 2.3 Data sources

The following databases will be searched from inception to the current date: Medline, Embase, the Cochrane Central Register of Controlled Trials, the Allied and Complementary Medicine Database, and the Cumulative Index of Nursing and Allied Health. We will also search 6 Korean medical databases (Online Archiving & Searching Internet Sources, Korean Traditional Knowledge Portal, Korean Studies Information Service System, KoreaMed, Korean Medical Database, and DBpia) and 3 Chinese databases including China National Knowledge Infrastructure (CNKI; i.e., China Academic Journal, China Doctoral Dissertations and Master's Theses Full-text Database, China Proceedings of Conference Full-text Database, and Century Journal Project), Wanfang, and VIP. Furthermore, we will conduct nonelectronic searches of conference proceedings and our own articles. The search strategies that will be applied to the Medline database is presented in Online Supplements 1. Similar search strategies will be applied to the other databases. Study selection will be documented and summarized in a PRISMA-compliant flow chart (http://www.prisma-statement.org).

#### 2.3.1 Types of studies

Prospective randomized controlled trials will be included if they are randomized studies of Suoquan as the sole treatment or as an adjunct to other treatments, as well as if the control group received the same treatment as the intervention group. Trials comparing Suoquan with any type of control intervention will also be included. No language restrictions will be imposed. Hard copies of all articles will be obtained and read in full.

#### 2.3.2 Types of participants

This study will include patients who were diagnosed with NE before the age of 18. We will exclude patients with NE secondary to urogenital system abnormalities, diabetes, diabetes insipidus, epilepsy, sleepwalking, and adverse drug reactions.

#### 2.3.3 Types of interventions

Interventions of any formulation (i.e., decoction, tablets, capsules, pills, powders, and extracts) of Suoquan will be included. In addition to the original form of Suoquan, we will also include formulas with additions or subtractions. The review will include only studies using Suoquan prescribed by traditional East Asian medicine practitioners. No limitations will be placed on the number, dosage, or duration of treatment.

#### 2.3.4 Data extraction and quality assessment

Two authors (YBL and HLL) will perform the data extraction and quality assessment using a predefined data extraction form (see Online Supplements 3–5). Any disagreement among the authors will be resolved through discussion among all of the authors. When the data are insufficient or ambiguous, HLL will contact the corresponding authors by e-mail or telephone to request further information or clarification. The risk of bias will be assessed using the assessment tool for the risk of bias from the Cochrane Handbook V.5.1.0, which includes random sequence generation, allocation concealment, blinding of the participants and personnel, blinding of the outcome assessments, incomplete outcome data, selective reporting, and other sources of bias.^[18]^ Our review will use “YBL” and “HLL” to indicate the results of the assessments, where “YBL” indicates a low risk of bias, and “HLL” indicates that the risk of bias was unclear or high. Disagreements will be resolved through discussion among all of the authors. When disagreements regarding selection cannot be resolved through discussion, an arbiter (H LL) will make the final decision.

### 2.4 Data collection and synthesis

#### 2.4.1 Outcome measures

##### 2.4.1.1 Primary outcomes

Number of wet nights per week

Improved effectiveness including total treatment efficacy (i.e., the number of patients whose NE symptoms improve)

##### 2.4.1.2 Secondary outcomes

Quality of life as measured using a validated questionnaire

Change in symptoms (e.g., urgency)

### 2.5 Information related to Suoquan usage

Pattern type of response, based on TKM or TCM theory

Range of Suoquan dosage in each study

Duration of treatment

Details of formula compositions

### 2.6 Data synthesis

Differences between intervention and control groups will be assessed. For continuous data, we will use the mean differences (MDs) with 95% confidence intervals (CIs) to measure the treatment effects. We will convert other forms of data into MDs. In cases of outcome variables with different scales, we will use the standard mean difference (SMD) with 95% CIs. For dichotomous data, we will present the treatment effects as relative risks (RRs) with 95% CIs. We will convert other binary data into RR values.

We will use the GRADEpro software of Cochrane Systematic Reviews to create a Summary of Findings table. When disagreements regarding the selections cannot be resolved through discussion, an arbiter (HLL) will make the final decision.

All of the statistical analyses will be conducted using the Cochrane Collaboration's Review Manager (RevMan) software V.5.3.5 for Windows. For studies with insufficient information, we will contact the corresponding authors to acquire and verify the data whenever possible. When appropriate, we will pool the data across studies for a meta-analysis using fixed or random effects.

### 2.7 Unit of analysis issues

For crossover trials, the data from the first treatment period will be used. For trials in which more than one control group was assessed, the primary analysis will combine the data from each control group. Subgroup analyses of the control groups will be performed and each patient will be counted only once in the analyses.

### 2.8 Addressing missing data

Intention-to-treat analyses that include all of the randomized patients will be performed. For patients with missing outcome data, carry-forward of the last observed response procedure will be used. The individual patient data will be sought from the original source or the published trial reports when the individual patient data are initially unavailable.

### 2.9 Assessment of heterogeneity

We will use a random- or fixed-effects model for the meta-analysis in accordance with the results of data analysis. *I*^2^ tests will be used to evaluate the heterogeneity of the included studies, and *I*^2^ >50 will be considered indicative of high heterogeneity. When heterogeneity is observed, we will conduct subgroup analyses to explore the possible causes.^[19]^

### 2.10 Assessment of reporting bias

If a sufficient number of the included studies (at least 10 trials) are available, we will use funnel plots to detect reporting bias.^[20]^ However, funnel plot asymmetries do not necessarily imply publication bias; therefore, we will attempt to determine the possible reasons for any asymmetries, such as small-study effects, low methodological quality, and true heterogeneities in the included studies.^[20,21]^

## 3 Discussion

In alternative medicine, Suoquan pills have been widely used in East Asia since ancient times for the treatment of NE. Suoquan was first described in *Introduction to Medicine* (*Yixuerumen*) by Li Chan in the Ming Dynasty (1368–1644).^[22]^ Suoquan is a mixture of Alpinia oxyphylla Miq., Dioscorea opposita Thunb., and Radix Lindera, prepared at a ratio of 1:1:1, and is used to warm kidney yang and expel cold while relieving frequent urination by stopping leakage.^[23–25]^ In South Korea, Suoquan is widely used for ameliorating urinary tract symptoms, such as NE and frequent or urgent urination.^[26]^ Various forms of Suoquan are available, such as pills, capsules, tablets, and decoctions. All types of Suoquan will be included in this study. So far, several reports have been published on the therapeutic effects of Suoquan on NE. However, no systematic reviews of the effects of Suoquan formulas on NE have been published. This systematic review will summarize recent evidence related to the effectiveness of Suoquan formulas in the treatment of the symptoms of NE. The review will be useful to practitioners and patients who suffer from NE.

## Author contributions

YBL and HLL conceived the study, developed the study criteria, searched the literature, analyzed the data, and wrote the protocol. YBL, HLL, and JAL conducted the preliminary search. All authors have read and approved the final manuscript.

**Conceptualization:** Ju Ah Lee.

**Funding acquisition:** Soobin Jang.

**Investigation:** Hye Lim Lee.

**Methodology:** Ju Ah Lee.

**Supervision:** Hye Lim Lee.

**Writing – original draft:** Yoo Been Lee.

**Writing – review & editing:** Hye Lim Lee, Soobin Jang.

## Supplementary Material

Supplemental Digital Content
