# Supplementary material for: Herbal medicine (Suoquan) for treating nocturnal enuresis: A protocol for a systematic review of randomized controlled trials
Source: Medicine (Baltimore). 2018 Apr 27;97(17):e0391. doi: 10.1097/MD.0000000000010391 (PMC5944475; doi:10.1097/MD.0000000000010391)
Supplement: Supplemental Digital Content [file medi-97-e0391-s001.docx]

Supplement 1. Search strategy

#1 Search "nocturnal enuresis"[Title/Abstract]

#2 Search "enuresis"

#3 Search "Nocturnal Enuresis"[Mesh]

#4 Search (#1 OR #2 OR #3)

#5 Search "Chinese medicine"[Mesh]

#6 Search "Kampo medicine"

#7 Search "Korean medicine" OR "Traditional Korean medicine"

#8 Search ((Plant Extracts"[Title/Abstract]) OR "Herbal Medicine"[Title/Abstract] or "herbal*")) OR (("Plant Extracts"[Mesh]) OR "Herbal Medicine"[Mesh]

#9 Search ‘Suoquan' OR 'Chukchunwhan' [All Fields]

#10 #5 OR #6 OR #7 OR #8 OR #9

#11 #4 AND #10
